# Noninvasive Hemoglobin Level Prediction in a Mobile Phone Environment: State of the Art Review and Recommendations

**DOI:** 10.2196/16806

**Published:** 2021-04-08

**Authors:** Md Kamrul Hasan, Md Hasanul Aziz, Md Ishrak Islam Zarif, Mahmudul Hasan, MMA Hashem, Shion Guha, Richard R Love, Sheikh Ahamed

**Affiliations:** 1 Department of Electrical Engineering and Computer Science Vanderbilt University Nashville, TN United States; 2 Department of Computer Science Marquette University Milwaukee, WI United States; 3 Department of Computer Science Stony Brook University Stony Brook, NY United States; 4 Department of Computer Science & Engineering Khulna University of Engineering & Technology Khulna Bangladesh

**Keywords:** noninvasive hemoglobin, smartphone-based hemoglobin, hemoglobin level from image and video

## Abstract

**Background:**

There is worldwide demand for an affordable hemoglobin measurement solution, which is a particularly urgent need in developing countries. The smartphone, which is the most penetrated device in both rich and resource-constrained areas, would be a suitable choice to build this solution. Consideration of a smartphone-based hemoglobin measurement tool is compelling because of the possibilities for an affordable, portable, and reliable point-of-care tool by leveraging the camera capacity, computing power, and lighting sources of the smartphone. However, several smartphone-based hemoglobin measurement techniques have encountered significant challenges with respect to data collection methods, sensor selection, signal analysis processes, and machine-learning algorithms. Therefore, a comprehensive analysis of invasive, minimally invasive, and noninvasive methods is required to recommend a hemoglobin measurement process using a smartphone device.

**Objective:**

In this study, we analyzed existing invasive, minimally invasive, and noninvasive approaches for blood hemoglobin level measurement with the goal of recommending data collection techniques, signal extraction processes, feature calculation strategies, theoretical foundation, and machine-learning algorithms for developing a noninvasive hemoglobin level estimation point-of-care tool using a smartphone.

**Methods:**

We explored research papers related to invasive, minimally invasive, and noninvasive hemoglobin level measurement processes. We investigated the challenges and opportunities of each technique. We compared the variation in data collection sites, biosignal processing techniques, theoretical foundations, photoplethysmogram (PPG) signal and features extraction process, machine-learning algorithms, and prediction models to calculate hemoglobin levels. This analysis was then used to recommend realistic approaches to build a smartphone-based point-of-care tool for hemoglobin measurement in a noninvasive manner.

**Results:**

The fingertip area is one of the best data collection sites from the body, followed by the lower eye conjunctival area. Near-infrared (NIR) light-emitting diode (LED) light with wavelengths of 850 nm, 940 nm, and 1070 nm were identified as potential light sources to receive a hemoglobin response from living tissue. PPG signals from fingertip videos, captured under various light sources, can provide critical physiological clues. The features of PPG signals captured under 1070 nm and 850 nm NIR LED are considered to be the best signal combinations following a dual-wavelength theoretical foundation. For error metrics presentation, we recommend the mean absolute percentage error, mean squared error, correlation coefficient, and Bland-Altman plot.

**Conclusions:**

We addressed the challenges of developing an affordable, portable, and reliable point-of-care tool for hemoglobin measurement using a smartphone. Leveraging the smartphone’s camera capacity, computing power, and lighting sources, we define specific recommendations for practical point-of-care solution development. We further provide recommendations to resolve several long-standing research questions, including how to capture a signal using a smartphone camera, select the best body site for signal collection, and overcome noise issues in the smartphone-captured signal. We also describe the process of extracting a signal’s features after capturing the signal based on fundamental theory. The list of machine-learning algorithms provided will be useful for processing PPG features. These recommendations should be valuable for future investigators seeking to build a reliable and affordable hemoglobin prediction model using a smartphone.

## Introduction

Hemoglobin (Hb) abnormalities cause several blood diseases, and lead to fatal and chronic health problems, including heart attack, stroke, and pregnancy complications [1]. When an adequate Hb blood level (men≥13 g/dL, women≥12 g/dL) is not maintained, the disorder complicates the function of the major organs (eg, kidney, brain, and heart) that require oxygen [2]. Anemia, a common Hb disorder, may be caused by blood loss, which is mostly a chronic condition (as occurs with menstruation), decreased red blood cell (RBC) production associated with iron and other nutritional deficiencies, and increased RBC destruction [3,4]. The central role of Hb is to maintain physiologic homeostasis, and the high frequencies of Hb abnormalities make assessment of this parameter a daily clinical activity.

Approximately 5.6% of the US population is anemic and 1.5% of the population has moderate to severe anemia [5]. Sickle cell diseases (SCD) cost more than US $1.5 billion annually in the United States [6]. Globally, blood disorders and associated complications affect more than 5 million people. In Africa, approximately 250,000 babies are born with SCD every year [7] and 1.62 billion people are affected by Hb-related abnormalities worldwide [8]. A reliable, affordable, and user-friendly solution is crucial to assess the Hb status of a large population. Clinical assessment of Hb typically involves the cyan-methemoglobin method, which is considered to be reliable. However, this invasive process has several limitations, including that the diagnostic devices are not portable, results are not immediately available, and the entire process is expensive. Thus, an Hb disorder diagnosis based on an invasive method is not a perfect solution, especially for people in low- and middle-income countries [9,10]. With available medical facilities, frequent invasive testing is also less convenient due to pain, anxiety, and infections [11]. A recent study estimated the cost for a complete blood count (CBC) test in Bari, Puglia, Italy, with approximately 1,000,000 inhabitants, to be US $3.14, resulting in a total cost of US $560,000 in 2018. Considering the entire national territory of Italy, the estimated cost will be more than US $20 million per year for public hospitals for outpatients. However, the laboratory costs for other cases, including hospitalized patients and private clinic patients, will be much higher than this previous estimation in Italy [12]. These multiple circumstances indicate the reasonable importance of a noninvasive point-of-care (POC) method for Hb measurement.

**Figure 1:**
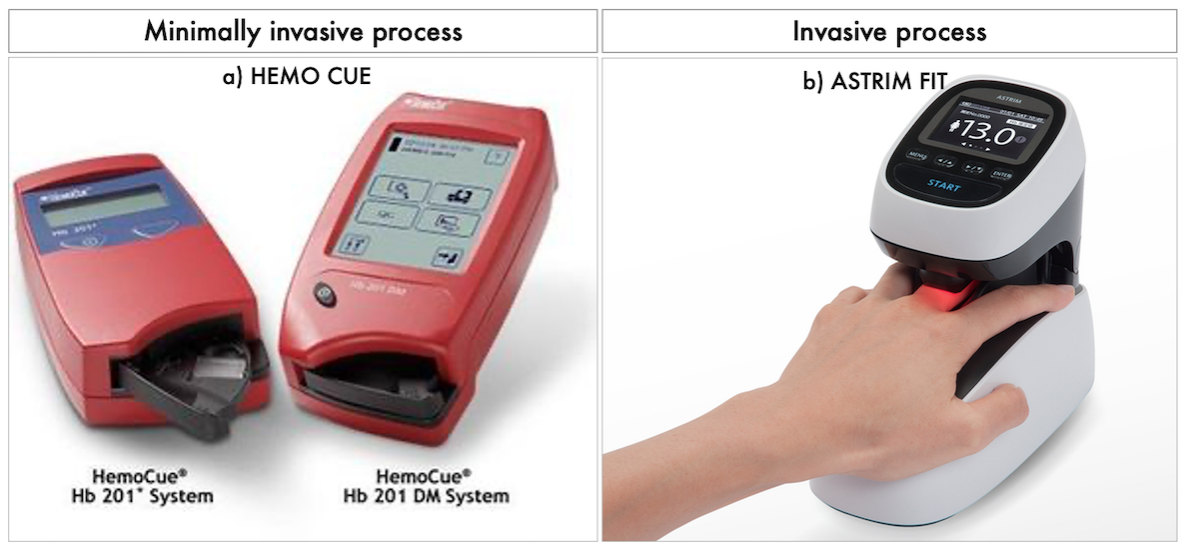
Point-of-care tools for minimally invasive and noninvasive hemoglobin measurement: (a) Hemo Cue, and (b) Astrim-Fit. (These two photos are licensed under CC BY-ND).

Commercially available noninvasive POC tools for Hb measurement (Figure 1) are already available [13-16], but have one or more of the following limitations: (1) challenging data collection methods, (2) complex data analysis and feature extraction processes, (3) lack of affordability and portability, and (4) lack of user-friendliness with costly external modules [17]. Smartphone-based solutions are emerging owing to their multifaceted benefits. Recent Hb level assessments use the signal captured from human body locations such as the fingertip [18], nail beds [19], and lower eyelid area [20]. The smartphone’s built-in sensors, additional attachments, signal processing methods, and machine-learning algorithms offer major advantages for Hb level estimation. However, most of these components (devices and data collection sites) vary among studies assessing noninvasive methods for Hb level estimation. Therefore, it is important to investigate what, how, and why these components play a vital role in Hb calculation.

Accordingly, in this study, we investigated invasive, minimally invasive, and noninvasive approaches to address the following research questions: (1) How is the signal captured by a smartphone camera from a body site? (2) What issues hinder the smartphone-captured signal for building a noninvasive diagnostic tool? (3) How are a signal’s features calculated considering a fundamental theory? (4) What machine-learning algorithms are used to develop a smartphone-based POC diagnostic app?

This study addressed the details of measuring Hb noninvasively. The paper is organized according to the functional components of a noninvasive Hb measurement system. The Methods section describes these components and briefly details current invasive and minimally invasive methodologies for Hb estimation. A list of noninvasive methods is discussed in detail, assessing the challenges and opportunities of smartphone-based solutions. In the Results section, we describe several sensors and signal processing methods that are currently available to process captured signals from different body sites and produce features to apply machine-learning algorithms. We further discuss the most common machine-learning algorithms, including ordinary least squares, multiple linear regression (MLR), partial least square regression (PLSR), and support vector machine regression (SVR). In the Discussion section, we provide several recommendations for the development of a POC tool using a smartphone and propose lighting sources to improve the measurement accuracy levels. Finally, we note our contributions to and limitations of this field.

## Methods

### Overview of Hb Estimation Methods

**Figure 2:**
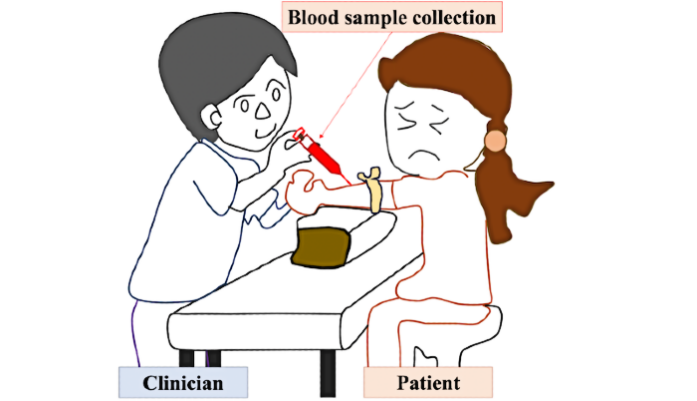
Collecting patient's blood sample for doing invasive hemoglobin diagnosis.

**Figure 3:**
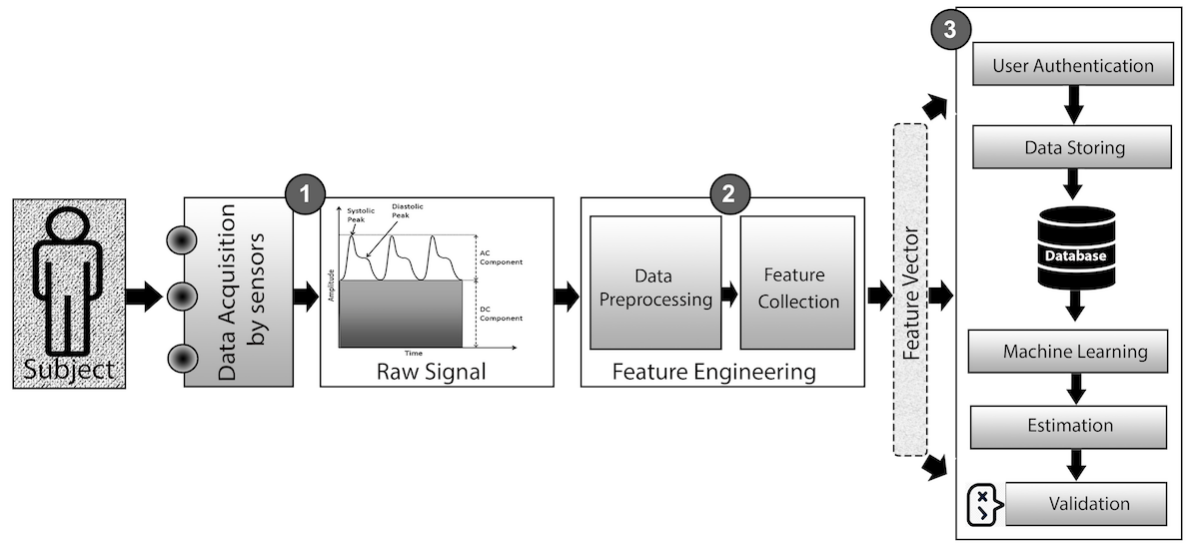
Phases involved in a noninvasive hemoglobin measurement system.

Hb level measurement is a blood diagnosis process to determine the concentration of Hb in the blood. Clinicians measure Hb in several ways, although the invasive (blood sample collection) approach remains the most common. Invasive processes involve the addition of various chemicals to a blood sample and then optical variations are calculated using spectroscopic data to measure the Hb level (Figure 2). By contrast, a noninvasive (without blood sample collection) approach involves data obtained from image sensors [21], spectroscopic information, and output of a photoplethysmographic (PPG) sensor to calculate the Hb level (Figure 3). In addition, a minimally invasive process requires only a couple of drops of blood to calculate Hb, and then collects image and spectra-based information from the blood sample for an estimation. Such minimally invasive techniques are comparatively less painful and have fewer complications in collecting sample data.

### Invasive and Minimally Invasive Processes

Smartphone-based solutions have appeared in recent years for invasive and minimally invasive blood Hb level measurement. In these cases, smartphones follow the characteristics of a spectrometer. For example, Edwards et al [22] built a smartphone-based G-Fresnel spectrometer that works within a wavelength range of 400-1000 nm. The G-Fresnel spectrometer showed a mean error of 9.2% in Hb level measurement in phantom tissue studies. A tungsten halogen lamp illuminates the liquid tissue phantom of human Hb and sends the diffusely reflected light to the G-Fresnel smartphone spectrometer. Although the smartphone-based spectrometer opens a new research horizon toward developing a portable and affordable solution, the use of liquid phantoms may not be an appropriate approach for layered biological tissues. Living tissue has both oxy- and deoxy-Hb molecules, and the phantom’s Hb is saturated mostly with oxygen. Therefore, in vivo studies seem to be more appropriate for developing a reliable smartphone-based solution to measure Hb levels.

A color-based POC system developed by Tyburski et al [23] was built with an inexpensive, disposable, and standalone device that consisted of two parts: a cap and a body. By capillary action, blood automatically fills the entire sample tube of the body. The cap is then placed into the body, which is prefilled with the reagent solution. After 60 minutes, the blood initiates a redox reaction and the solution shows a stable color change. Using a color scale sticker or with the optional smartphone app after capturing an image of the solution, the Hb level was measured in 238 patients. The sensitivity of the visual interpretation and smartphone analysis of this POC device was 90.2% and 91.1%, respectively, and the specificity was 83.7% and 79.2% respectively. However, this minimally invasive approach suffers from the limitation of the reagent’s expiration date, quality of the cap and body of the device, captured image quality and resolution, and identification of an exact Hb level by a visual scale.

Visual image-based approaches have also been introduced for Hb measurement. Glycated Hb (HbA_1c_), which provides information about an average sugar level for the last 4 months, can be measured using a paper-based system and a smartphone, which will help to capture an image of a drop of blood. Using this colorimetric process, Siva et al [24] applied image-processing techniques to investigate the pixel color intensity values and correlated the level of Hb with HbA_1c_ [24]. Siemens Healthineers developed and commercially launched a blood diagnosis device named Aina that can be attached to a smartphone for determining levels of Hb, HbA_1c_ glucose, and a lipid profile [25].

A chromatography paper-based test was developed that involves a mixture of blood and Drabkin reagent based on bloodstain images digitized with a portable scanner to quantify Hb levels [26]. This process may be accomplished with a smartphone camera sensor by developing a mobile app and analyzing the captured image. For continuous Hb monitoring, a sensor that can calculate Hb levels is implanted in the body, which requires replacement every 3-4 days due to enzyme depletion and membrane contamination [27]. In this implanted system, a wire has to be attached to the patient’s body to transmit signals [28].

In these invasive and minimally invasive systems, drawing blood from a vein involves insertion of a needle, which can cause some discomfort, pain, numbness, or a shocking sensation to patients, with subsequent itching or burning at the collection site. These procedures are often traumatic for children and people with mental disabilities. This situation is further exacerbated for patients with needlephobia, a medical condition affecting approximately 10% of the global population [29].

### Noninvasive Process

Noninvasive systems are usually composed of the three main functional components shown in Figure 3: (1) a data acquisition sensor that captures a raw biological (eg, image or spectral) signal; (2) a feature engineering unit that preprocesses the signal and calculates features from the signals; and (3) an Hb level estimation system, which generally incorporates different layers of user authentication, data storage, prediction model usage, machine learning, and result validation [21]. The user authentication and Hb estimation phase depend on the device, internet availability, user data, and prediction model.

Before elaborating on the smartphone-based noninvasive approaches, we investigated spectroscopy-based techniques with near-infrared (NIR) spectroscopy (NIRS), because these methods have received considerable attention for the noninvasive measurement of blood Hb, oxygenation, pH, hematocrit, and glucose levels [30]. We investigated the lighting sources used in various NIRS methods to determine which NIR lights are most useful in calculating an Hb level noninvasively. The light sources used in a spectra-based investigation may enhance the chance of obtaining accurate Hb information using a smartphone camera to which lights can be attached as an external device.

External lights (ie, NIR lights) are required when a smartphone has no support to sense blood Hb noninvasively in living tissues. One noninvasive method for measuring Hb flow involves analyzing the response of an NIR spectrometer that monitors variations in the absorption of NIR light in the arm, followed by calculating the changes in deoxy-Hb and oxy-Hb concentrations using six wavelengths: 797.5, 802.5, 831.2, 848.7, 866.5, and 907.8 nm [31]. A strong correlation (*R*^2^=0.95) between Hb values calculated by venous occlusion PPG and NIRS was determined. Using the PPG signals under eight wavelengths (ranging from 600.22 nm to 1000.60 nm), Yi et al [32] improved the accuracy of dynamic spectrum extraction and analyzed transmitted light through the fingertip of 220 subjects. They developed a calibration model between the dynamic spectrum data and Hb levels, obtaining a correlation of *r*=0.86 and a root mean square error of prediction of 8.48 g/L. Although the estimated Hb levels were accurate and precise, closely matching clinical requirements, there is an opportunity to involve a more rational calibration set selection process and further improvements of the instrument’s signal to noise ratio (SNR). Again, this solution should involve a portable and low-cost instrument.

Table 1 summarizes other spectra-based Hb level measurement processes, which vary in terms of the ranges of light wavelength, input signals, and acquisition devices. In most cases, investigators have used an expensive spectrometer for data collection. Among these spectra-based studies, the most commonly used spectral wavelengths have been 850 nm, 940 nm, and 1070 nm. Investigators have also employed specialized devices to capture PPG signals from the data collection sites such as the finger, hand, and earlobe.

**Table 1:** Summary of spectra-based techniques proposed for noninvasive hemoglobin measurement.

Reference	Wavelength (nm)	Comparator	Signal	Participants (N)
Yi et al [32]	600-1100	Hematology analyzer (Pentra 60; ABX; France)	PPG^a^	220
Rochmanto et al [33]	670, 940	Sysmex-KN21	PPG	78
Desai et al [34]	530	Pronto-7, Hemocue Hb analyzer	PPG	10
Kavsaoglu et al [35]	660, 905	Hemocue Hb-201TM	PPG	33
Kim et al [36]	400-700	Standard CBC test	Photon	32
Nirupa et al [37]	624, 850	Prototype	PPG	69
Ding et al [38]	600-1050	LED^b^ and photodiode	Spectra	119
Bremmer et al [39]	350-1050	Ocean Optics DH-2000	Spectra	8
Timm et al [40]	600-1000	LED	PPG	48
Fuksis et al [41]	760-940	IR^c^ LEDs	Spectra	—^d^
Pothisarn et al [42]	660, 940	Analyzer oximetry	Light	—
Nguyen et al [43]	940	Radical 7, XE-2100	Pulse	41
Jeon et al [44]	569, 660, 805, 880, 940, 975	Hemoglobin cyanide method	Pulse	129
Jakovels [45]	500-700	White LED	Spectra	—
Timm et al [46]	600-1400	OxyTrue Hb	Spectra	1008
Wang et al [47]	500-700, 1300	Masimo Pronto 7, RGB CMOS camera	PPG	32
Suzaki et al [48]	600, 625, 660, 760, 800, 940, 1300	K1713-09 Hamamatsu Photonics, Co-oximeter	Light	—
Al-Baradie et al [49]	670	Hemo Cue	PPG	10

^a^PPG: photoplethysmography.

^b^LED: light-emitting diode.

^c^IR: infrared.

^d^—: information not provided.

## Results

### Smartphone as a POC Tool

A smartphone-based POC tool as a potential alternative to invasive clinical blood testing is rapidly attracting attention because of the advantages of availability, user-friendliness, and easy attachability to different biosensing devices. The combination of a smartphone and an external device can offer a reliable and affordable POC tool for remote health monitoring. Moreover, the enhanced computing ability, sensing capability, portability, and wide availability of smartphones have propelled this development.

Approximately 56% of US adults and more than 2.5 billion people worldwide are currently using smartphone devices. Multiple critical issues have been addressed in employing smartphones for clinical measurement, including for physiological parameter estimation [50-52], noninvasive Hb diagnosis [53,54], and blood glucose measurement [55,56]. Several studies have shown a higher level of performance in some biomedical applications where a smartphone plays a pivotal role in measuring blood oxygenation, Hb, glucose level, cholesterol, and antibody levels (see Multimedia Appendix 1). The most frequently used smartphones are developed by Apple, Samsung, Motorola, Google, HTC, Sony, and Asus, where the camera sensor is used to capture videos or images. The accuracy level was deemed to be reliable in each of the studies listed in Multimedia Appendix 1 [50,51,54,55,57-64]. Data commonly captured by a smartphone were obtained from two main body sites: the fingertip and eyelid.

### Finger-Based Analysis

The average width of a human index finger is 14 mm, including the bone (∼6 mm), tissue, dermis (∼3 mm), epidermis (∼1.5 mm), and nail-plate (∼1 mm) [65]. As a data collection site, the finger is frequently chosen for several reasons: it is easy to place on a smartphone, it is less sensitive than the eyelid, and it is easy to control. In most cases, the finger pulp area is illuminated using either the phone flashlight or external light sources to obtain the pulsatile information of blood in this area. Reflectance and transmittance oximetry, based on the light source’s position, have been applied to the fingertip area using a smartphone to estimate Hb levels. For example, SmartHeLP [53], HemaApp [66], and Hb Meter [67] determinations have used smartphone camera sensors to capture image or videos. In these studies, various lengths of fingertip videos were recorded with different smartphones, and each video frame was analyzed pixel-wise by separating the red, green, and blue (RGB) pixel intensities. Hasan et al [53] subdivided each frame into 10×10 similar sized blocks, separating RGB pixel intensities, and generating time-series information on each block over all frames. They also applied an artificial neural network to estimate Hb levels based on training data of 75 subjects. The gold-standard Hb levels ranged from 7.6 to 13.5 g/dL, and a rank-order correlation of 0.93 was obtained between model-predicted and gold-standard Hb levels. Based on the pixel information from the group of blocks, the most significant region of interest was determined to be close to the smartphone’s flashlight. Although RGB pixels were explored in this study, only the red pixel information was employed for development of the prediction model. In addition, the presence of extreme (lower and higher) levels of Hb was limited.

**Figure 4:**
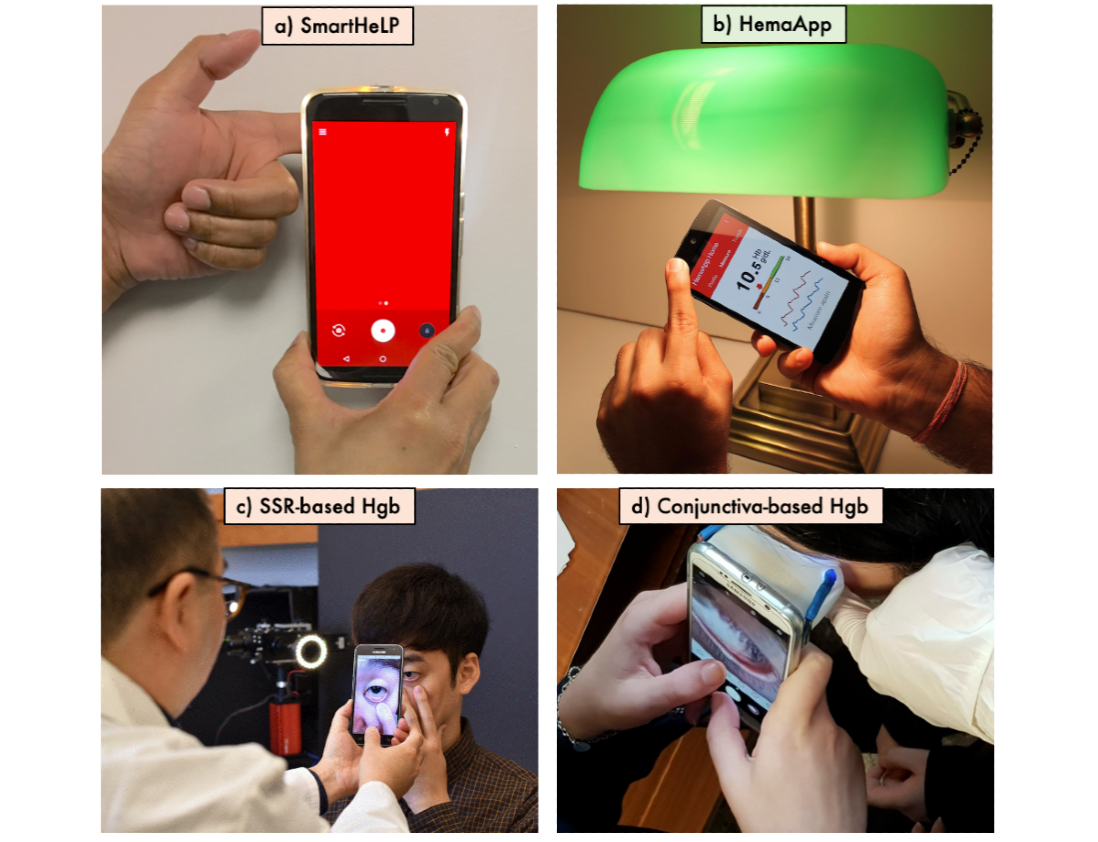
Smartphone-based point-of-care tools for noninvasive hemoglobin measurement using fingertip and eyelid images. (a) SmartHeLP [53], b) HemaApp [66], (c) SSR-based Hgb [20], and (d) Conjunctiva-based Hgb [80]. The images are presented with permissions.

Similarly, Wang et al [66] evaluated fingertip videos using three different hardware embodiments, in which the first embodiment included a white flash and infrared emitter, the second embodiment incorporated an incandescent lamp with a white flash and infrared emitter, and the third embodiment was made by a white flash and custom infrared light-emitting diode (LED) array (Figure 4) [66,68-70]. The external lighting sources, a combination of incandescent and NIR LEDs, resulted in better estimation with an error of 1.26 g/dL and correlation of *r*=0.82 compared with the other two embodiments. In this case, they captured 15-second-long fingertip videos from 31 subjects, generated pulsatile signals, and extracted RGB time-series waveforms for each video. Additional features, including peak and trough, were calculated from each time-series dataset, and SVR was applied to estimate the level of Hb for each user. In this study, the analyzed Hb levels ranged from 8.3 g/dL to 15.8 g/dL, which were compared with those estimated using Masimo Pronto. Although HemaApp showed greater accuracy than Masimo Pronto, HemaApp was not tested on various types of devices (eg, smartphone) and lighting sources. HemaApp used the bulb to receive light of about 1000 nm, and the age of the light bulb impacted the efficiency. In addition, the effect of ambient light in this study was significant. To make HemaApp more versatile, the prediction model requires upgrading on the training data with more subjects.

In another report, four LED lights with different wavelengths, photodiodes, and a microcontroller unit were used to capture a finger’s PPG signal, enabling calculation of the ratio between alternating current (AC) and direct current (DC) signals of the PPG, and estimation of the Hb level, which was transferred to a smartphone through Bluetooth [71]. The microcontroller analyzed the PPG signal using the exponential moving average and then linear regression was applied to calculate the Hb level of 30 subjects, with a root mean square error of 1.53 g/dL. However, the light setting requires correct illumination for precise Hb estimation and the effect of different skin pigmentation is yet to be tested in this system.

A human fingernail, with about 1 mm thickness on average, is comprised of keratin protein, which is translucent [72]. Fingernails have been studied since they allow for easy data capture and they are relatively easy to control [73]. Mannino et al [19] analyzed the images of a fingernail bed captured by a smartphone-based app to investigate critical information for noninvasive Hb level measurement. In this study, an Apple iPhone 5s captured the fingernail bed images (with the camera flash both on and off) from 337 participants who provided blood samples for a standard CBC test. Multilinear regression with a bisquare weighting algorithm was applied to build a prediction model from the nail bed’s image parameters and standard laboratory reports. Although the smartphone app measured the Hb level within 2 gm/dL with a bias of 0.2 gm/dL in 100 patients, and showed a good correlation (*r*=0.82) compared with CBC reports, the system suffers from a limitation of automated region of interest selection.

Most of the finger-based studies for Hb level estimation considered reflectance oximetry, in which the smartphone camera and the light source were on the same side. However, transmittance oximetry has been rarely applied for finger-based data collection in estimating Hb levels. There is an opportunity to investigate the finger as a data collection site by applying transmittance oximetry, in which light from the finger’s dorsal area is sent to the pulp area, because peak absorption of the human melanin pigment occurs at around 335 nm [74], whereas tissue has low absorbance (translucent) in the red and NIR regions, and prior studies indicated that NIR light could penetrate more than 1-2 cm [75].

### Palpebral Conjunctiva

The palpebral conjunctiva, the lower eyelid area of the eye, has received considerable attention as a measurement site because the microvessels in this area are clearly visible and melanocytes are not present [76]. Reflectance spectroscopy has been applied to capture data from the eyelid area [77]. Digital photography and spectral data of lower eyelid images or spectral data converted from an RGB image were applied in several studies to measure Hb levels in a noninvasive manner.

Recently, Park et al [20] introduced a smartphone-based solution that converts an RGB image captured by a smartphone’s camera sensor into a virtual hyperspectral image. The need for additional equipment such as an attachment with a smartphone to capture the spectral response was avoided by generating a conversion matrix (T) to transfer a regular image to a spectral image. The generated spectral image was defined as virtual spectra, which were used to train an Hb prediction model. In this study, a wide range of Hb values were calculated in clinical settings and compared with the estimated Hb levels, and Bland-Altman analyses showed reliable performance. Although three different smartphones were used to collect the data, the testing on other smartphones, creation of a more extensive dataset with a wide range of Hb levels, and inclusion of patients with a variety of possible confounding medical conditions are required for further evaluation of this approach.

Selim et al [77] developed a solution based on a lower eyelid image captured by a commercially available Sony DSC-F1 digital camera with a charge-coupled device (CCD), exposing the palpebral conjunctiva. To minimize the effect of ambient light, a gray card was placed close to the eye, and the region of interest was selected from both the eyelid and gray image manually. Invasively measured Hb, using an automated cell counter (SE 9500, Sysmex Corporation, Japan), was compared with the estimated Hb of 117 subjects’ eyelid images, demonstrating a Pearson rank-order correlation coefficient of 0.6. However, the prediction algorithm was not verified with other light sources, compared with a gold-standard test, checked according to the variation in oxygen saturation, or tested in outdoor settings.

Dimauro et al [78] attached an enclosed macrolens with a smartphone to capture a close and high-resolution image of an eyelid with precise focus. The image was segmented using the SLIC Superpixels algorithm, a region of interest was selected for feature extraction, the erythematous was calculated for the CIE-Lab color space, and the k-nearest neighbor classification algorithm [79] was applied to the eyelid image data captured from 102 participants. Applying the Random Oversampling Examples (ROSE) balancing algorithm, they found reliable prediction of the Hb level using conjunctiva images.

Digital images of the palpebral conjunctiva can provide information to measure the level of Hb in a noninvasive manner [80]. Anggraeni et al [57] built a regression model using the digital images of 20 participants’ eyelids along with white paper images captured at the same time by Asus ZenFone 2, and estimated the Hb concentration, which correlated highly with clinically measured Hb levels (*r*=0.92). Among the three color pixels of a palpebral conjunctiva image, the red color intensity showed better performance than the green and blue pixel intensities in this study. However, specific software is required for image analysis, and the region of interest needed to be selected for enhancing the precision level.

In addition to conjunctiva images, Rojas et al [81] developed Selienemia, a smartphone and cloud-based platform, using RGB, ISO files, and exposure of images of the tongue, and built a curve-fitting model applying logistic regression and a neural network algorithm. The tongue images provided a better result (sensitivity 91.89% and specificity 85.18%) than the conjunctiva-based prediction model (sensitivity 91.89% and specificity 70.34%) when tested on 64 patients. However, the training model was built on a population in which most of the participants were young (mean age of 22.6-31.6 years) and extreme levels of Hb were rarely observed in this group (mean 10.6-14.8 g/dL). Establishing a controlled environment and standardized images for Selienemia is challenging.

Although noninvasive devices can capture accurate blood Hb values, their application can be cumbersome and limit users, since the devices have to be attached and oriented correctly, and must be operated with expertise. Access to expensive noninvasive devices for Hb diagnosis is not a practical solution in many low- and middle-income countries. Since the number of smartphone users in the world is estimated at about 6 billion [82], discussion about noninvasive methods should involve user-friendly and cost-effective solutions developed using a smartphone; however, more details of the sensors used are required. In the following sections, we discuss several sensors and signal processing tools.

### Sensors

Sensors translate a physiological signal into machine-accessible data that allow for measurement of physical properties of the human body by collecting physiological signals from one or multiple body sites, including the skin [45], fingertip [66], lip [83], and eye conjunctiva [84]. A machine-learning algorithm with the features generated from a sensor’s signal can be used to build a prediction model to estimate Hb levels.

Table 2 lists the different types of sensors that have been used to capture physiological data to estimate Hb levels noninvasively. Most of these sensors are based on image, PPG signal, and optical data. Some of the sensing devices were built by the research team, whereas others used off-the-shelf hardware such as a smartphone, PPG device, or spectrometer.

**Table 2:** Summary of different sensors, signal types, and body sites used for hemoglobin level measurement.

Reference	Device	Sensor	Signal	Body part
Kavsaouglu et al [35]	Hemocue Hb-201	PPG^a^	Light	Finger
Kim et al [36]	Spectrometer, quartz-tungsten-halogen source	Optical	Spectra	Conjunctiva
Nirupa et al [37]	Prototype	PPG	Light	Finger
Ding et al [38]	LED^b^ and photodiode	Optical	Spectra	Finger
Timm et al [40]	InGaAs photodiode	Optical	Spectra	Finger
Pothisarn et al [42]	Analyzer oximetry	Optical	Light	Finger
Nguyen et al [43]	XE-2100, Masimo Radical 7	Fluorescence and optical	Pulse	Finger
Jeon et al [44]	Hardware prototype	Optical	Pulse	Finger
Jakovels et al [45]	Nuance 2.4	Optical	Spectra	Skin
Timm et al [46]	Hemocue	Optical	Spectra	Finger
Wang et al [47]	Masimo Pronto 7, RGB^c^ CMOS^d^ camera	Image	PPG	Fingertip
Kamrul et al [53]	Smartphone camera	Image	PPG	Finger
Wang et al [66]	Smartphone camera	Image	PPG	Finger
Kuestner et al [85]	Modified pulse oximeter, Coulter STKS Monitor	Optical	Spectra	Finger, ear or toe
Lamhaut et al [86]	Hemocue 201+, Radical-7	Optical	Spectra	Finger or ear
Jakovels et al [87]	RGB CMOS	Optical	Spectra	Arm
Miyashita et al [88]	R1-25 and R2-25a	Optical	Spectra	Finger
Li et al [89]	AvaSpec HS1024x58TEC-USB2	Optical	Spectra	Finger
Frasca et al [90]	Hemocue 301, Siemens RapidPoint 405, Sysmex XT 2000i	Optical	Spectra	Finger

^a^PPG: photoplethysmography.

^b^LED: light-emitting diode.

^c^RGB: red, green, blue.

^d^CMOS: complementary metal oxide semiconductor.

Optical sensors, as a type of photometric device, capture the optical signal from an external source such as an LED, laser, or lights of different spectra [91]. Photodiodes are primarily used as optical sensors, which are made of indium gallium arsenide (InGaAs) and indium-phosphor. In some cases, optical sensors contain an embedded amplifier that can select different wavelengths (500-1600 nm) of the signal. A complementary metal oxide semiconductor (CMOS) is a sensor that converts photons to electrons for digital processing, which is used in smartphones, digital video cameras and digital CCTV cameras, astronomical telescopes, scanners, barcode readers, robots, and optical character recognition systems. As high-end smartphone devices include CMOS camera sensors, we can collect data using a smartphone in data collection. In addition to RGB, a CMOS sensor, RGB LED ring-light illuminator, and orthogonally orientated polarizers can be used to capture images, where multiple images under different light sources may carry rare information [87]. The reason is that CMOS chips, with PPG light-capturing cells, pick up the photons at different wavelengths and translate them into electrons, which are converted by digital-to-analog converters into pixels of various colors [92]. The CCD sensor, a light-sensitive integrated circuit, can convert each image pixel into an electrical charge, and has a high degree of sensitivity that can generate an image even in low-light conditions [93].

InGaAs, an alloy of indium arsenide and gallium arsenide, is another type of infrared sensor used in photodiodes. As a faster response, an InGaAs photodiode is preferred in most studies since these photodiodes have shown higher quantum efficiency [94]. An InGaAs photodetector may also be useful for noninvasively monitoring the Hb concentration and oxygen saturation [95]. A silicon photomultiplier is a solid-state photon detector that can count every single photon, is small in size, of low cost, able to detect low light, and is quantum-efficient [96]. Several studies have used a silicon photomultiplier to build embedded systems to detect PPG signals in both reflective and transmittance modes [97].

A PPG sensor captures an optical response from the microvascular bed of a fingertip, and is used for arterial, venous, and respiratory measurements. Recently, optical PPG sensor data were used for noninvasive Hb measurement [98]. A PPG device can identify the finger motion with a motion detector and can work with more than one wavelength. Compared with data captured by an electrocardiogram machine, a PPG device reliably (*R^2^*=0.93) calculated the heart rate of 170 subjects [99].

Sensing through an electrical-sensing device costs additional money, which complicates the use of such systems. By reducing the number of electrical sensors through leveraging the smartphone’s camera sensor, an image or video signal can be collected from a body site, and these data can be processed to generate machine-readable signals and features, and then machine-learning algorithms are applied to build an Hb prediction model. Smartphone-captured data, either image, audio, or video, should be preprocessed using signal processing techniques.

### Signal Processing

Smartphone-recorded signals captured from a body site are attenuated by different unintentional issues such as movement, external noise, and motion artifacts. As part of preprocessing, smartphone-captured data, image, or video are processed using OpenCV library, which generates time-series signals [100] that can be applied to Fourier series analysis on a cycle-by-cycle basis. To remove high-frequency noise in the signal, the data can be filtered using smoothing filters such as Savitzky-Golay smoothing, Butterworth, and Gaussian filters. A cycle-by-cycle Fourier series analysis could reduce the measurement error of the signal from 37% to 3% [101].

The Savitzky-Golay data smoothing filter uses a least-squares polynomial approximation by fitting a polynomial to an input dataset, and evaluates the resulting polynomial at a single point, maintaining the shape and magnitude of the waveform peaks while smoothing the waveform [102].

Biological signals, which are nonstationary as they tend to change over time, can be passed through wavelet transformation for noise reduction and signal enhancement [103]. Stationary wavelet transform was applied to PPG signals, and the wavelet transforms modulus maxima was used to reduce motion artifacts, resulting in an 87% reduction in heart rate estimation error, 76% reduction in heart rate variability estimation error, and 66% reduction in instantaneous heart rate error [104]. A continuous wavelet transform can be used to determine the accurate position of the peak and trough of a PPG signal [105]. However, wave transform has limited capability in restoring corrupted PPG signals for both heart rate and pulse transmit time measurements [106]. There are also more advanced techniques derived from wave transform such as synchrosqueezing transform that have been used to process PPG signals [107].

Independent component analysis (ICA) can separate the additive non-Gaussian subcomponents of a multivariate signal [108]. As motion artifacts in a PPG signal are derived from independent sources, these can be separated using ICA. ICA can also be used to separate the effect of ambient light and other sources of interference. Kim et al [109] used a combination of ICA and block interleaving with low-pass filtering to reduce motion artifacts in PPG signals. Holton et al [110] compared ICA with principal component analysis, another source separation technique, with respect to their effectiveness in PPG signal recovery from video recordings, and found that ICA produced the most consistent result.

A Butterworth filter, as a maximally flat filter, makes the frequency response of a signal as flat as possible in the passband [111]. By applying the Butterworth filter, high-pass, low-pass, or band-pass filter, a PPG signal can be processed as an authentication method of a PPG biometric [112]. With the Butterworth filter, using both low-pass filtering and wavelet transform, motion artifacts can be removed from PPG data, monitor blood pressure, and identify wrong peaks [113-115].

A biological signal captured by a smartphone introduces noise due to uncontrolled data collection processes, which results in a low SNR. To remove the motion artifact, a Butterworth filter [113], singular value decomposition [116], adaptive filtering [117], Fourier series analysis [118], ICA [109], and principal component analysis [119] have been most commonly used. More than one technique should ideally be used to reduce the motion artifact based on the generated signal’s pattern, noise level, sources, environment, and acquisition process. After cleaning, features of the signal are calculated to apply machine-learning algorithms to build a prediction model.

### Machine-Learning Algorithms

#### Definition

A machine-learning algorithm trains a machine to learn and apply acquired knowledge in predictions. Most of the current Hb prediction models use machine-learning algorithms. Although these algorithms could be used in any type of diagnostic system, we here present a list of machine-learning algorithms that are commonly used to assess Hb levels noninvasively (Table 3).

**Table 3:** Summary of machine-learning algorithms for noninvasive hemoglobin measurement.

Reference	Algorithms	Performance measures
Demauro et al [12]	kNN^a^ classifier	*r* and *R*^2^
Yi et al [32]	Difference accumulation	*r*, RMS^b^
Kavsaouglu et al [35]	CART^c^, LSR^d^, GLR^e^, MVLR^f^, PLSR^g^, GRNN^h^, MLR^i^, SVR^j^	MSE^k^, *R*^2^, RMSE^l^, MAPE^m^, IA^n^
Nirupa et al [37]	Linear regression	MSE, *R*^2^
Ding et al [38]	BP-ANN^o^ and PCA^p^	*r*
Bremmer et al [39]	LLS^q^ fit	*r*
Jeon et al [44]	MLR, PLSR	MSE, *R*^2^, *r*
Jakovels et al [45]	Regression analysis	Gaussian analysis
Timm et al [46]	Regression	BAA^r^
Wang et al [47]	Linear regression	RMSE
Wang et al [66]	SVR	*r*, BAA
Lamhaut et al [86]	Linear regression	*r*, BAA, *P* value, bias, precision
Miyashita et al [88]	Linear regression	*r* and BAA bias plot
Li et al [89]	PLSR	R
Frasca et al [90]	Regression, BAA	MSE, *r*, RMSE, *R*^2^, BAA
Kamrul et al [18,53]	PLSR and ANN^s^	R

^a^kNN: k-nearest neighbor.

^b^RMS: root mean square.

^c^CART: classification and regression trees.

^d^LSR: least-squares regression.

^e^GLR: generalized linear regression.

^f^MVLR: multivariate linear regression.

^g^PLSR: partial least-squares regression.

^h^GRNN: generalized regression neural network.

^i^MLR: multiple linear regression.

^j^SVR: support vector regression.

^k^MSE: mean square error.

^l^RMSE: root mean square error.

^m^MAPE: mean absolute percentage error.

^n^IA: index of agreement.

^o^BP-ANN: backpropagation artificial neural network.

^p^PCA: principal component analysis.

^Q^LLS: linear list squares.

^r^BAA: Bland-Altman analysis.

^s^ANN: artificial neural network.

#### MLR

With a similar strategy of more than one simple linear regression, MLR aims to model the relationship between two or more explanatory variables and a response variable. The simple linear regression estimates the relationship between a dependent variable *Y* and an explanatory variable *X* using the equation *Y_i_*=*β*_0_+*β*_1_*X_i_*+ε_i_, where *β*_0_ is the intercept and *β*_1_ is the slope of the line, and the error ε_i_ is considered to have a mean value of 0. By contrast, MLR has *p* explanatory variables. In this case, the relationship between *Y* and *X* is represented by the following equation:

*Y_i_* = *β*_0_ + *β_1_X_1i_* + *β_2_X_2i_* + *β*_3_*X_3i_* +...+ *β_p_X_pi_* + ε_i_,

where *β*_1_ to *β_p_* are the coefficients. Using this equation, MLR uses the features of a signal as an observation (row) of *X* and the target value. For example, the clinically measured Hb level is stored in *Y* to build an Hb prediction model [35].

#### PLSR

Multiple factors, greater than the number of observations, have been analyzed by PLSR in several studies for Hb level estimation [120,121]. PLSR calculates a few latent factors among other factors that may be responsible for most of the variation in the target or response variable. PLSR names the latent variable as *T* or *X* scores and defines the response variables as *U* or *Y* scores. The *X* scores with a direction in the factor space explain the factor variation, even when a strong relationship with *Y* scores is lacking. In PLSR, the *Y* scores maintain the variation of predicted *Y* and provide data regarding the change in *U*. The greatest advantage of PLSR is that both *X* and *Y* scores are used to determine a correlation, which helps to build a reliable prediction model [122].

#### SVR

SVR is a well-known regression technique for the dimensionality problem, which finds the best hyperplane that separates a class/group with maximum distance using support vectors as a set of critical points. The optimization function is given as follows [123]:

1/2 ||*w*||^2^

Subject to,

*y_i_* – (*w*_i_*x*_i_) – *b* < *e*

(*w_i_x_i_*) + *b* – *y_i_* < *e*

SVR uses kernels, linear or nonlinear, to create a hyperplane that preserves maximum margins among the data. One of the popular kernel functions is the radial basis function, which has been used to estimate noninvasive Hb levels [35,66,124].

### Measurement Techniques

An Hb prediction model developed by applying a machine-learning algorithm from estimated Hb levels requires a performance test with a gold-standard (clinically measured) Hb value. Performance measurement, based on comparison of estimated with clinically measured values, is generally achieved by calculating the goodness of fit (*R*^2^), correlation coefficient (*r*), mean absolute percentage error (MAPE), Bland-Altman plot, mean absolute error, and mean squared error (MSE) in data analysis, as follows.


MAPE: 
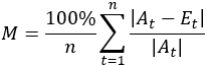



where *A_t_* is the actual value or gold-standard measurement, *E_t_* is the estimated value, and *n* is the number of measurements or observations. MAPE is used in the majority of performance measurements because it is easy to explain and understand and does not depend on scale.

If *Y_i_* denotes the *i*th target value and *Ŷ_i_* denotes the estimated value of *Y_i_*, then the formula for the MSE considering the dependent variable *Y* with *n* elements is:



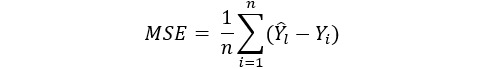



The correlation coefficient (*r*) demonstrates how strongly two measurement methods are linearly related. The value of *r* is between –1.0 and +1.0; if *r* is +1.0 or –1.0, then strong linear relationships are indicated. The formula for Pearson correlation is given by [125]:



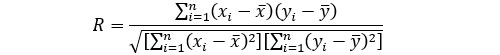



where *n* is the sample size, *x_i_, y_i_* are the sample points, 
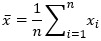
 is the sample mean, and 
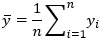
 is the target mean value.

The Bland-Altman plot is used to estimate a limit of agreement (LOA) between two quantitative measurements. In general, it is common to compute the 95% LOA between two measurement processes. The Bland-Altman plot thus represents the difference between the two measurement methods against the mean value.

## Discussion

### Summary

We investigated several invasive, minimally invasive, and noninvasive methodologies involving a smartphone for data collection, presentation, and transmission processes toward the development of a noninvasive Hb measurement tool. The diverse methodologies across studies included data collection processes, signal processing techniques, feature selection processes, prediction model development algorithms, and performance measurement techniques. Based on these insights, we provide a list of recommendations to develop a smartphone-based noninvasive Hb level estimation tool, which are organized below to answer the research questions on how to capture a signal using a smartphone camera from a body site, address several issues that add noise in the smartphone-captured signal, calculate the features of a signal following a fundamental theory, and apply machine-learning algorithms for the development of an Hb prediction model.

### Body Site Selection for Signal Acquisition

The recommended optimal data collection sites on the body are the palpebral conjunctiva, because of easy access to the microvasculature, and the fingertip, because of the ease of control and access. In the eyelid area, most data collection processes involve digital photography [77,84,126] or reflectance spectroscopy [127,128]. Although most studies demonstrate how to capture an image accurately, perform spectral measurement, and maintain the data collection site motionless during data collection, there is a chance that some of the measurements may include noise from other unintentional activities such as eye blinking, eye sensitivity to the light, breathing, loss of control of the eyelid, or a limited exposed eye area. While using the smartphone camera or external camera to capture an eyelid image, the user can attach a fixed object to the smartphone (eg, mirror) and the image can be captured with a mobile app, in which the boundary of the eyelid area must be visible so that users capture the eyelid image from a fixed distance. In this case, the secondary camera (or the camera on the screen side) of the smartphone is a good option since the user can see the app screen and the eyelid area on the smartphone’s screen, which may allow capturing an eyelid image without additional assistance.

The fingertip has several advantages as a data collection site. Fingertips are easily accessible, less sensitive to minor manipulations, and are generally easy to control. The approximate thickness from the dorsal to the ventral pad side of a finger is about 14 mm for adults. Fingernails with a translucent protein (keratin) can transfer NIR light, which can penetrate more than 1-2 cm [73,75]. Thus, NIR light in the finger tissue can work in both reflective and transmissive mode. Owing to the greater flexibility, we recommend a fingertip-based study over an eyelid-based approach.

### Response Calculation

**Figure 5:**
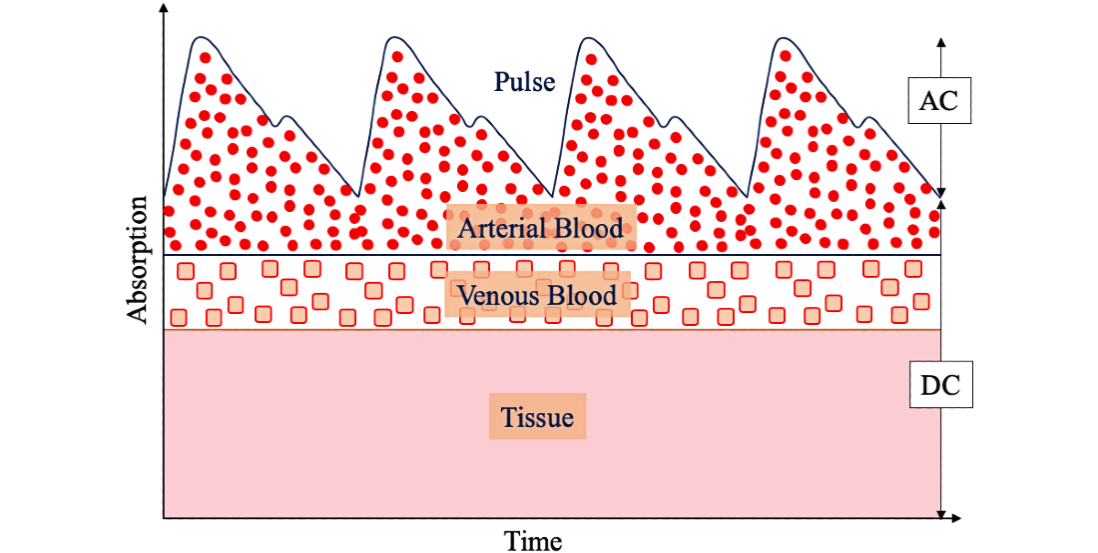
Light absorption changes for pulse, arterial, and venous blood, and living tissue. AC: alternating current; DC: direct current.

Fingertip tissues with arterial and venous blood contain light-absorbing components that can be recorded by PPG, an optical device that can be used to observe blood volume changes noninvasively. A PPG system is built with a light source to illuminate the tissue area (eg, finger) and a photodetector to capture the variation of light intensity. The intensity variations are observed due to the systole and diastole parts of the heartbeat. Thus, a PPG signal is derived from two parts: the dynamic part, defined as the AC signal, and the static part, defined as the DC signal (Figure 5).

The absorption of light by melanin and fat in the skin exhibits a significant response in the shorter wavelengths of light [129]. NIR light, with a wavelength range from 700 to 2500 nm, can penetrate the finger more efficiently than visible light. In this range, light can penetrate 1-2 cm in tissues. As suggested by the foregoing discussion, we recommend light sources of 850 nm or 940 nm, and camera (visible) light to assess the Hb response from a fingertip video. Since water starts showing a response at greater than 950 nm, we recommend using 1070-nm wavelength light to capture the plasma response from a video. The PPG signal captured under an NIR LED light source should be used for further processing. Since the low SNR may reduce the possibility of better PPG generation, selection, and feature extraction, we recommend utilization of laser diodes as a light source and developing a PPG generation algorithm using the dynamic spectrum method [89], ratio of the superimposition averaging template and pulse wave [130], optimized differential extraction method [131], and spectral difference coefficient and dynamic spectrum [132].

### Signal Preprocessing

Because the pressure of the fingertip pad on the smartphone camera and finger movement can alter the waveform of the PPG signal, a well-designed hardware system for securing the imaged finger needs to be developed [133,134]. Noise and artifacts can be further reduced with the use of filters such as moving average and adaptive filters that work with a reference signal [135]. The reference signals can be obtained from an additional transducer to identify finger movement [136]. Most physiological signals are nonstationary and change their properties over time. In this case, a wavelet transformation and the smoothed pseudo-Wigner-Ville distribution are recommended to improve the PPG signals [135]. The wavelet transform has been used as a common method of movement artifact reduction for PPG signals [137].

To identify the region of interest, HemaApp uses the center section of an image [66], Scully et al [58] used 50×50-sized image pixel intensities on the green channel, and Jonathan and Leahy [138] took a central region with a mean intensity value from 10×10 pixels for smartphone-based PPG generation. Based on these findings, we recommend subdividing an image into a 10×10 image block, generate a PPG signal on each block against all frames, and identify the best location to obtain the strongest PPG signal.

### Theoretical Foundations

The transmissive or reflective process captures the properties of a living tissue noninvasively [139]. The variation of this transmitted or reflected light depends on the shape, volume, and refractive index of Hb, and the angular distribution of scattered light, which characterizes the absorption properties of blood and tissue [140]. By analyzing these changes in optical scattering properties in tissues, a noninvasive solution for Hb estimation can be achieved.

According to the Beer-Lambert law, *I_o_*=*Ie*^−^*^αCD^*, where *I_o_* is the output light intensity, *I* is the incident light intensity, *α* is the light absorption coefficient, *C* is the concentration of a blood component, and *D* is the light path; the absorption of light is proportional to the concentration of a medium and the path length. A finger has three different absorptions for a given wavelength of light (*λ*) due to Hb, plasma (*P*), and the tissue (*T*). Therefore, the light absorption (under a given *λ*) by a finger is

*Io,λ* = *_Ie_*(*αHb*[*Hb*]+*αP*[*P*]+*αT*[*T*])(−*D*)

Following the above equation, the light response for the AC and DC value of a PPG can be given as:

*AC_λ_* = *_Ie_*(*α_Hb_*[*Hb*]+*αP*[*P*])(−*d*1)+(*αT*[*T*])(−*DT*)

*DC_λ_* = *_Ie_*(*α_Hb_*[*Hb*]+*αP*[*P*])(−*d*2)+(*αT*[*T*])(−*DT*),

where *d*_1_ is the path length for Hb and plasma during the AC signal, *d*_2_ is the path length for Hb and plasma during the DC signal, *d*=*d*_1_−*d*_2_, and *D_T_* is the path length for the tissue. We assume that the tissue has a stable response, and the ratio of the magnitude of AC and DC removes the effect of the tissue. Then, we can express the ratio between the AC and DC values as:

ACλ/ DCλ=e(αHb[Hb]+αP[P])(–d)

where *d* is the path length that affects only the Hb and plasma for *λ* wavelength of light. Taking the log of both sides of the equation, we can write:

lnAC_λ_/ DC_λ_=(α_Hb_[Hb]+α_P_[P])(–d)

The empirically measured absorption coefficient for each wavelength of light can help to solve the above equation. However, the system setup for fingertip video recording, lighting conditions, PPG generation from fingertip videos, and complex reflection properties of tissue require machine-learning regression techniques to calculate the ratio of Hb and plasma [54,66]. By incorporating multiple wavelengths of light and the respective responses, several studies have demonstrated reliable Hb prediction models, reducing the number of wavelengths to two with one Hb-sensitive wavelength and another plasma-sensitive wavelength. We recommend this dual-wavelength approach, in which the ratio of the responses captured by two different wavelengths of NIR lights has been applied in different investigations such as for blood Hb [35], skin blood supply assessment [141], oxygenation level [142], and glucose level [143] estimation. The CCD camera sensors can capture PPG signals similar to a pulse oximeter using a photodetector in the NIR range, with a light wavelength around 1000 nm [144]. Modern smartphone cameras have strong sensing capabilities for PPG imaging and volumetric changes in the arterial blood, which enable them to capture PPG signals using reflective or transmissive oximetry from the finger. After generating the PPG signal from the smartphone-based fingertip videos, the features can be calculated from each signal.

### PPG Feature Generation

**Figure 6:**
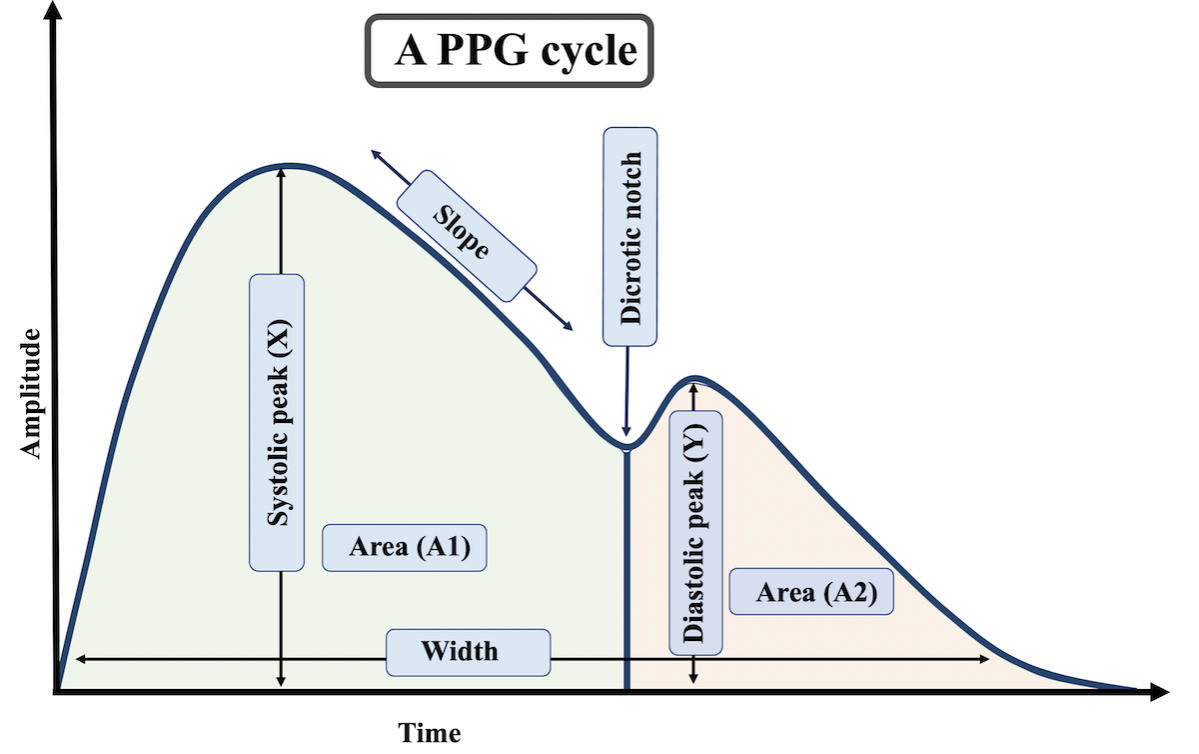
Multiple features collection from a photoplethysmogram signal.

Since a PPG signal reflects the movement of blood from the heart to the fingertip through the blood vessels, the characteristic parameters of a PPG signal may provide information on blood constituent levels. PPG features have been used in several studies, including those of hematocrit, oxygen saturation, pulse, and respiration [35,145-147]. Based on these insights, we recommend investigating multiple features from the PPG signal (Figure 6), including the systolic and diastolic peak, PPG rise time, pulse transit time, pulse shape, and amplitude [148].

The systolic peak, an indicator of the pulsatile changes in blood volume caused by arterial blood flow, is generated by the direct pressure wave coming from the left ventricle to the periphery of the body. The diastolic peak is a result of reflections of the pressure wave by arteries of the lower body [147]. The dicrotic notch is a small downward deflection between the systolic and diastolic point of a PPG cycle [149]. The pulse interval represents the relationship between the contribution that the wave reflection makes to the systolic arterial pressure and the reflected wave coming from the center [148]. The PPG shows blood movement, whereas the first derivative of the PPG signal indicates the velocity of blood in the finger [150]. Finally, the ratio of a peak value and the sample rate is denoted as the peak time.

The systolic amplitude, representing pulsatile changes in blood volume, can lead a machine-learning algorithm to correlate the pulsatile changes with blood constituent levels [151]. Delle et al [152] confirmed the inverse relationship between the middle cerebral artery peak systolic velocity and Hb levels. With the incoming arterial pulse in the systolic phase, the total light absorbance rises with the increase in arterial blood volume. The systolic increase can then be measured by subtracting the diastolic baseline absorbance from the systolic peak absorbance [153]. The relative augmentation allows us to capture these variations [66], and the inflection points can determine the minimum and maximum values of the PPG waveform [154]. By calculating the first and second derivatives of the PPG signals, the informative inflection points can be more accurately studied. The change in blood volume can be tracked by calculating the pulse interval and the ratio of different peak arrival times [155].

Finally, we recommend calculating the ratio of two PPG features captured under two different wavelengths of light (*λ_H_* and *λ_P_*). The ratio of two PPG signals’ feature values can be presented as follows:

R*_λ1_* (*λ*2)=PPG*_λ1_/PPG_λ2_*,

where, *R_λ_*_1_(*λ*_2_) is the ratio of the two PPG signals’ features, *PPG_λ_*_1_ is a PPG generated under the Hb-responsive light source, and *PPG_λ_*_2_ is a PPG calculated under a plasma-responsive light source. The ratio of the two PPG feature values represents the individual ratio between each feature value, which can then be applied to Hb level estimation.

### Dataset Balancing

In medical research, an imbalanced learning problem frequently occurs while solving a classification problem due to insufficient data of certain classes [12]. The imbalance condition can affect the prediction model. Therefore, suitable solutions are required to solve this problem. One strategy might be to alter the class distribution through data resampling (eg, oversampling with sample replacement). The newly generated data can remove the overfitting issues and improve the generalization ability. Class balancing can be achieved through the ROSE algorithm [156], which helps to relieve the severity of the effects of an imbalanced distribution of classes. SMOTE [157], which is based on an oversampling approach, can also be applied to solve this issue.

### Patient Evaluation Strategy

There are different types of users or patients worldwide of a smartphone-based POC solution for blood component measurement. Based on the availability of smartphones and the expertise of the user, two strategies can be adopted. The first strategy is for users living in low-resource settings, who can obtain the smartphone from a local clinic, pharmacy, village shop, or government office such as a municipality. Since the users are not experts in using the mobile app and face challenges in understanding the output of the blood report, a trained person can help the patient collect the fingertip video or capture an eyelid image to transfer to a cloud for further processing. In this case, the smartphone is safe to use without the risk of losing a device, sending wrong data, and obtaining misleading feedback from the cloud. This option is also cost-effective since many people can access the smartphone with minimum payment. The second strategy is for smartphone users who have some degree of mobile health literacy, confidence to capture data, and a better understanding of mobile apps. In these settings, the users capture data from fingertip videos or eyelid images by themselves and submit the data through the internet. In both contexts, users are also allowed to send their clinical blood test results through the mobile app to the cloud. These strategies will help researchers to build an updated prediction model based on the data stored on the cloud.

These recommendations can provide guidance for researchers in the area of noninvasive blood component measurement to develop smartphone-based POC tools with the support of mobile app development (user interface), cloud computers, and software and prediction model developers. The data collected by a smartphone can be transferred to a cloud via the internet where several steps are to be accomplished, such as authentication, schedule data to a job manager [158], apply an existing prediction model, update the model, and give feedback to the users with an estimated Hb level.

### Conclusions

**Figure 7:**
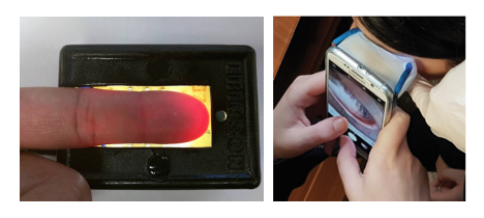
Recommended data collection tool design for (left) fingertip video capture and (right) an eyelid conjunctiva image.

As an increasingly widely available computing platform, the smartphone offers an alternative, noninvasive POC tool to traditional measurements of blood Hb. We recommend the fingertip as the data collection site for the optimal development of an accurate Hb prediction model due to its easy access, use of three different NIR lighting sources, specific signal processing techniques and feature selection methods, and region of interest selection methods. For fingertip-based data collection, a covered external NIR light source (ie, fully covered PPG device) can provide the best PPG signal from a smartphone video. The video should be captured with minimum presence of ambient light, as demonstrated by Hasan et al [159] (Figure 7, left). In addition, an eyelid conjunctiva image can be captured using a smartphone app installed on a head-mounted plastic passive viewer (Figure 7, right) [12,78]. These two data collection methods can provide practical applications because of their reliability, ease of use, and sustainable cost for a patient. Investigators need to consider the following issues before developing such a smartphone-based POC tool: (1) cost of the smartphone, external device, reagents if needed, training, internet, and cloud implementation; (2) other physiological features of the patient; (3) enabling multiple checks with a minimal cognitive load for the user; (4) storing the user’s location, sex, and age in the record; (5) keeping the external device as optional so that a user can run a diagnostic without the device; and (6) creating an external device that is cost-effective, easily attachable, properly fit with the finger, and user-friendly.

## Appendix

Multimedia Appendix 1 Summary of smartphone-based solutions offered in physiological parameter monitoring processes.

## Abbreviations

AC: alternating current
CBC: complete blood count
CCD: charge-coupled device
CMOS: complementary metal oxide semiconductor
DC: direct current
Hb: hemoglobin
HbA_1c_: glycated hemoglobin
ICA: independent component analysis
InGaAs: indium gallium arsenide
LED: light-emitting diode
LOA: limit of agreement
MAPE: mean absolute percentage error
MLR: multiple linear regression
MSE: mean squared error
NIR: near infrared
NIRS: near infrared spectroscopy
PLSR: partial least-squares regression
POC: point of care
PPG: photoplethysmography
RBC: red blood cell
RGB: red, blue, green
ROSE: Random OverSampling Examples
SCD: sickle cell diseases
SNR: signal to noise ratio
SVR: support vector machine regression
