# Supplementary material for: Noninvasive Hemoglobin Level Prediction in a Mobile Phone Environment: State of the Art Review and Recommendations
Source: JMIR Mhealth Uhealth. 2021 Apr 8;9(4):e16806. doi: 10.2196/16806 (PMC8063099; doi:10.2196/16806)
Supplement: Multimedia Appendix 1 [file mhealth_v9i4e16806_app1.docx]

Summary of smartphone-based solutions offered in physiological parameter monitoring processes.

| Reference | Purpose | Smartphone | Captured | Result |
| --- | --- | --- | --- | --- |
| Zaman et al [50] | 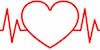 | iPhone 4/5/6 | 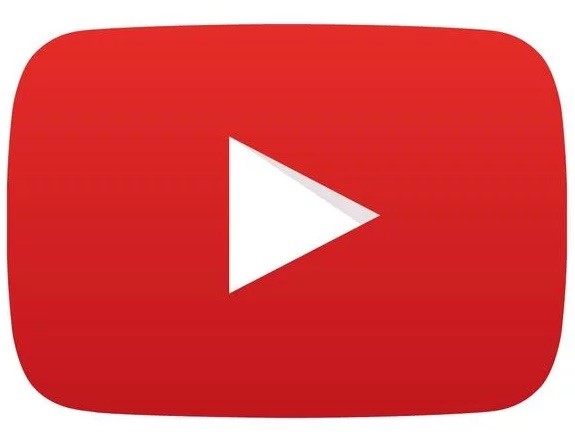 | SD=0.08 Hz |
| Peng et al [51] | 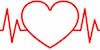 | HTC S510e | 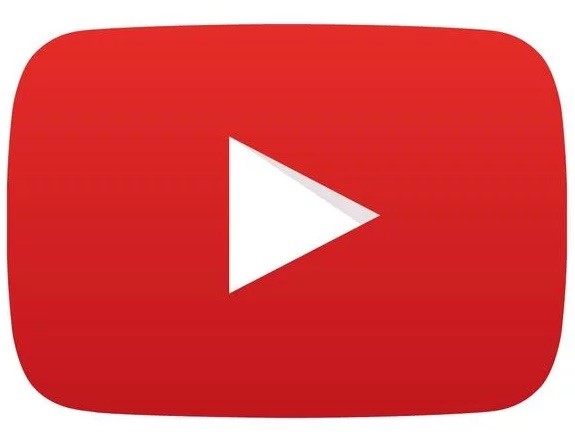 | *r>*0.7 |
| Wang et al [54] | 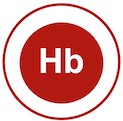 | Nexus 6p | 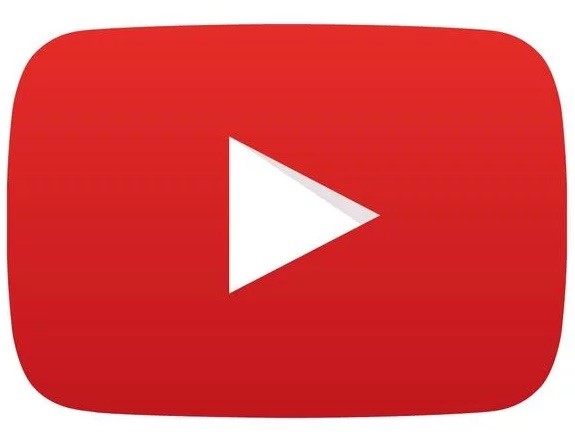 | *R*^2^=0.62 |
| Dantu et al [55] | 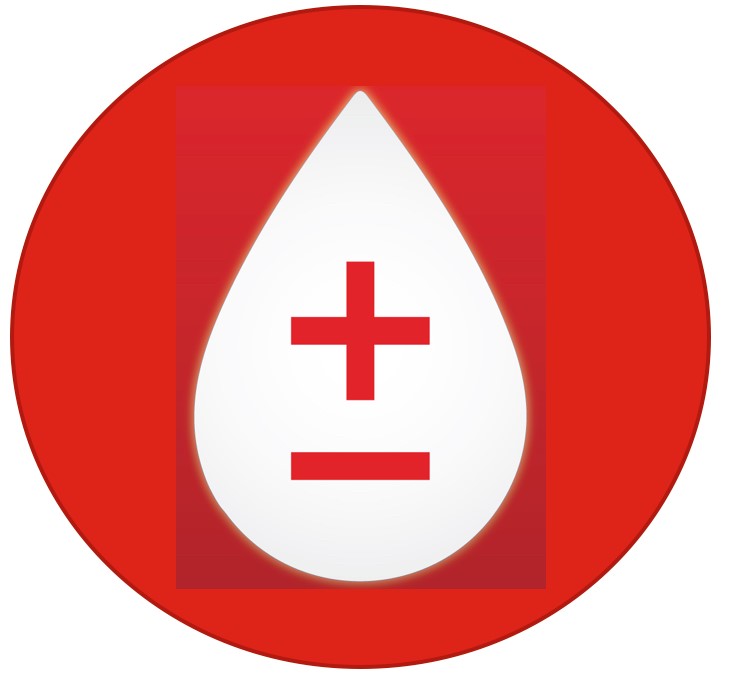 | HTC One X | 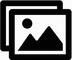 | *P*=.005 |
| Gregoski et al [153] | 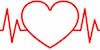 | Motorola Droid | 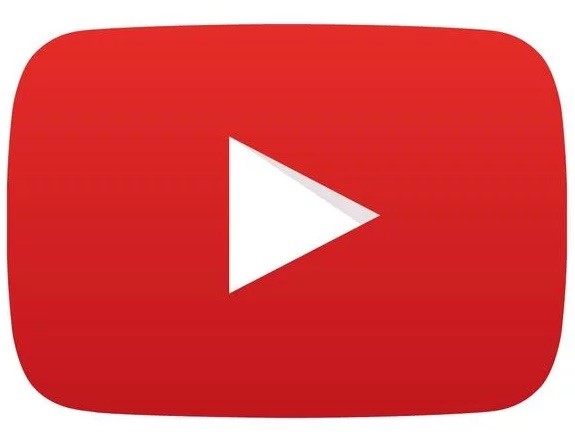 | *r*=0.95 |
| Scully et al [131] 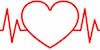 | 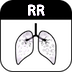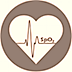 | Motorola Droid | 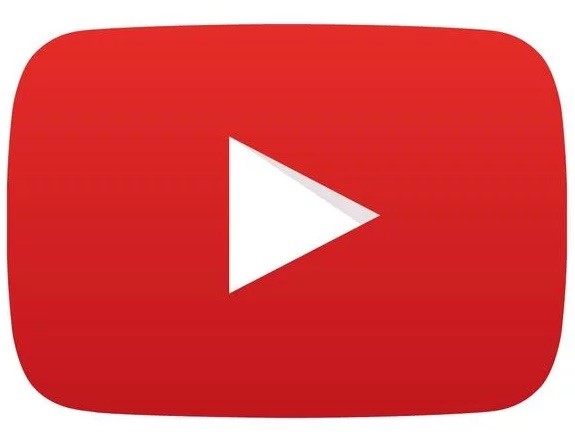 | 92.2 (SD 5.3) |
| Oncescu et al [154] | 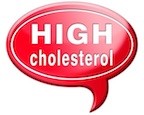 | iPhone | 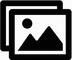 | error<5.5% |
| Liu et al [155] | 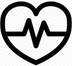 | PPG modules | FV & FT | *r*=0.86 (SD 0.06) |
| Singhal et al [156] | 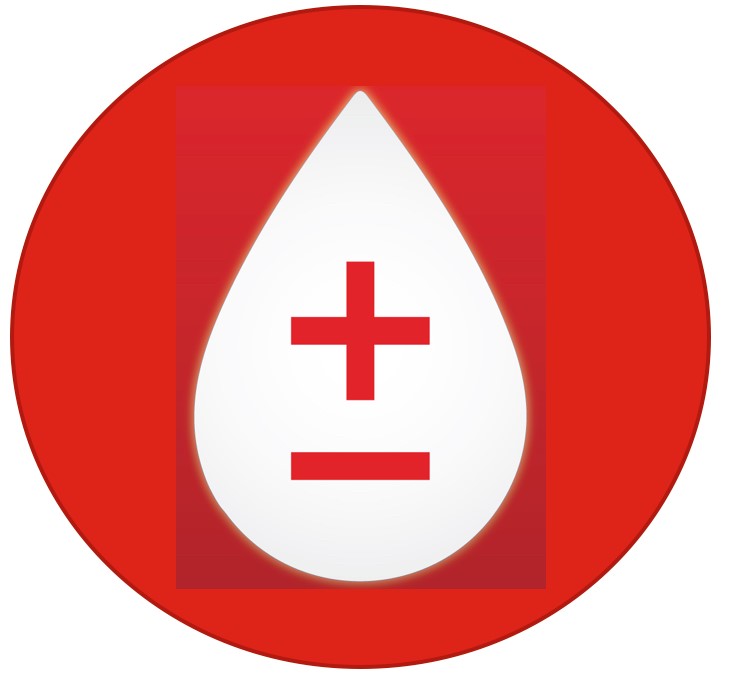 | Sony Xperia Arc LT15i | 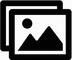 | *R*^2^=0.98 |
| Soni et al [157] | 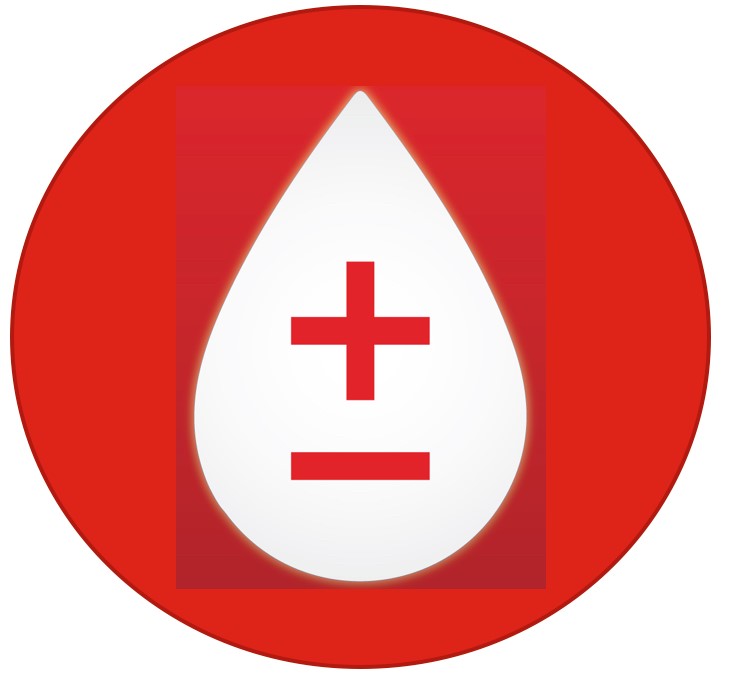 | Samsung Galaxy SIII | 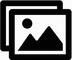 | *r*=0.44-0.94 |
| Arts et al [158] | 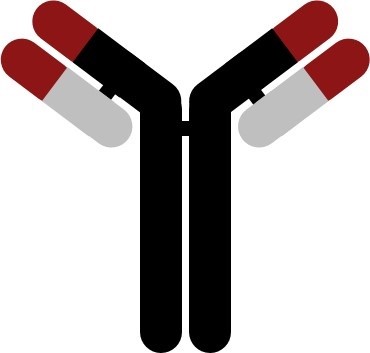 | Nokia Lumia 920 | 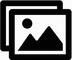 | *R*^2^=0.97 |
| Shin et al [159] | 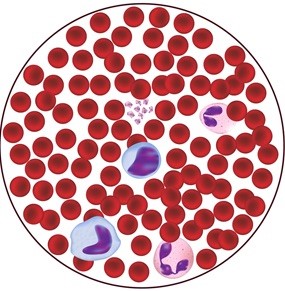 | Samsung Galaxy SII | 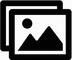 | *R*^2^>0.97 |
| Zhu et al [160] | 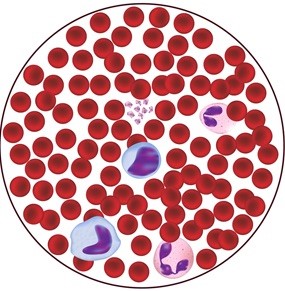 | Samsung Galaxy SII | 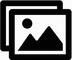 | *R*^2^=0.92, error<5% |
| Anggraeni et al [73] | 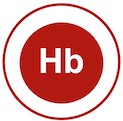 | Asus Zenfone 2 Laser | 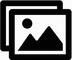 | *R*^2^=0.81 |
| Wu et al [162] | 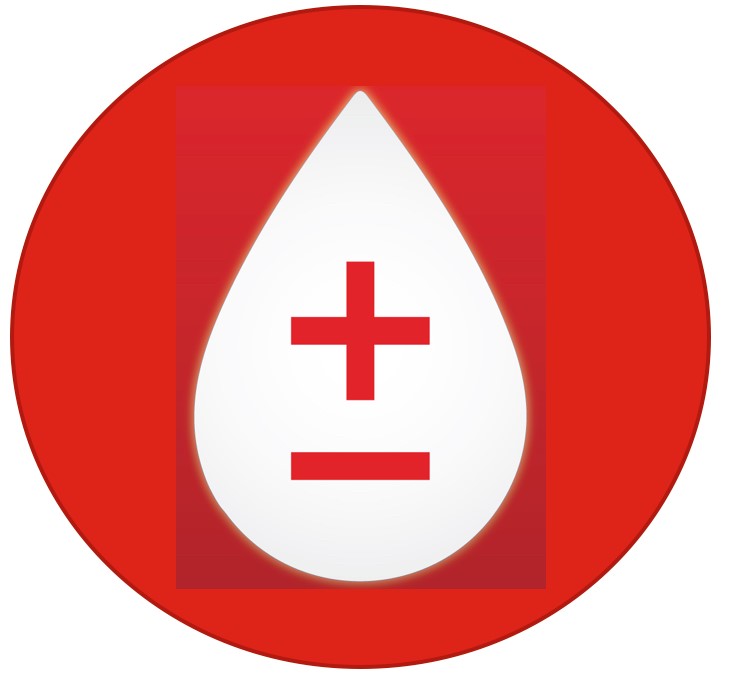 | iPhone 4 | 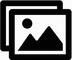 | *R*^2^=0.99 |
| Devad et al [163] | 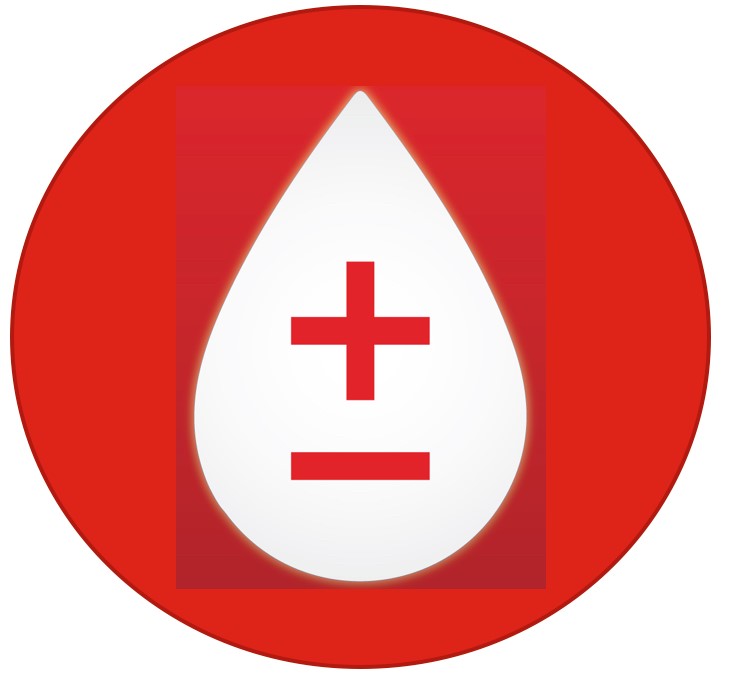 | Samsung | 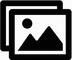 | *R*^2^=0.967 |
| Hasan et al [165] | Pain | Nokia X6 | 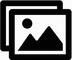 | Mean absolute error*=*2.9% |
| Hasan et al [166] | 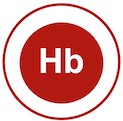 | Nexus 4 | 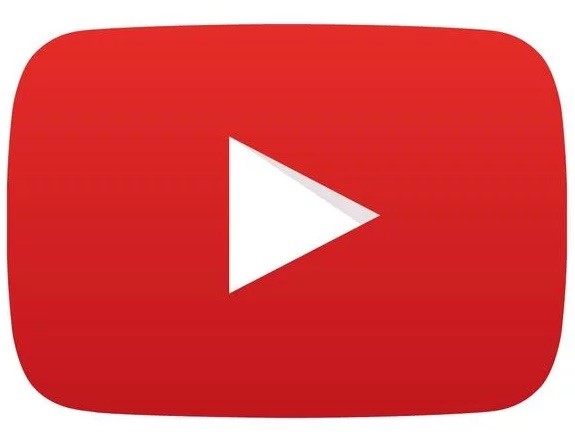 | *R*^2^=0.87 |

|  |
| --- |
| 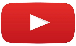 = Video, 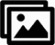 = Photo, 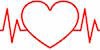=Heart Rate, 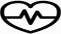 = Pulse transit time, 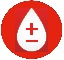 = Glucose, |
| 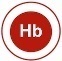 = Hemoglobin, 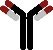 = Blood Antibodies, 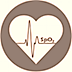 = Respiratory Rate, 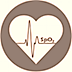 = SpO_2_, |
| 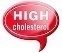 = Cholesterol, 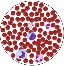 = Red/White Blood Cells, 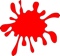 = Age of Blood Stain |
